# On Markov blankets and hierarchical self-organisation

**DOI:** 10.1016/j.jtbi.2019.110089

**Published:** 2020-02-07

**Authors:** Ensor Rafael Palacios, Adeel Razi, Thomas Parr, Michael Kirchhoff, Karl Friston

**Affiliations:** a The Wellcome Centre for Human Neuroimaging*,* University College London*,* Queen Square*, London* WC1N 3BG*,* UK; b Monash Institute of Cognitive and Clinical Neurosciences and Monash Biomedical Imaging*,* Monash University*,* Clayton*,* Australia; c Department of Electronic Engineering*,* NED University of Engineering and Technology*, Karachi,* Pakistan; d Department of Philosophy*,* Faculty of Law*,* Humanities and the Arts*,* University of Wollongong*, Wollongong* 2500*,* Australia

**Keywords:** Self-organisation, Markov blanket, Dynamical systems, Free energy, Active inference

## Abstract

Biological self-organisation can be regarded as a process of spontaneous pattern formation; namely, the emergence of structures that distinguish themselves from their environment. This process can occur at nested spatial scales: from the microscopic (e.g., the emergence of cells) to the macroscopic (e.g. the emergence of organisms). In this paper, we pursue the idea that Markov blankets – that separate the internal states of a structure from external states – can self-assemble at successively higher levels of organisation. Using simulations, based on the principle of variational free energy minimisation, we show that hierarchical self-organisation emerges when the microscopic elements of an ensemble have prior (e.g., genetic) beliefs that they participate in a macroscopic Markov blanket: i.e., they can only influence – or be influenced by – a subset of other elements. Furthermore, the emergent structures look very much like those found in nature (e.g., cells or organelles), when influences are mediated by short range signalling. These simulations are offered as a proof of concept that hierarchical self-organisation of Markov blankets (into Markov blankets) can explain the self-evidencing, autopoietic behaviour of biological systems.

## Highlight

• Computational treatment of biological self-organisation.
• Biological self-organisation requires emergence of boundaries, namely Markov blankets.
• Hierarchical self-organisation entails emergence of Markov blankets at multiple scale.

## 1 Introduction

There is growing interest in the role of Markov blankets and associated partitions in understanding self-organisation– and the accompanying self-evidencing that arises from Bayesian mechanics (Friston, 2019). A key aspect of this self-organisation is the hierarchical decomposition of Markov blankets of Markov blankets. This notion has emerged in the literature at several levels; ranging from conceptual analyses in the context of ethology and evolution (Allen, 2018; Clark, 2017; Friston et al., 2015; Kirchhoff et al., 2018; Pellet and Elisseeff, 2008; Ramstead et al., 2018), through to the emergence of multicellular organisms (Kuchling et al., 2019) to the implicit renormalisation group that furnishes a particular perspective on (quantum, statistical and classical) mechanics (Friston, 2019; Friston et al., 2014).

However, despite the potential importance of these conceptual and mathematical analyses, no one has yet provided a proof of principle that Markov blankets of Markov blankets can emerge using numerical analyses. In this paper, we report such a proof of principle by illustrating the emergence of blankets of blankets under the unitary principle of (variational) free energy minimisation. We frame this in terms of self- organisation or pattern formation in cells, to emphasise the simplicity and biological plausibility of the underlying dynamics – although this framing is more by analogy than any detailed consideration of inter-and intracellular communication. Our primary aim was to show that hierarchal compositions of Markov blankets of Markov blankets can emerge from gradient flows on variational free energy, under an appropriate generative model.

In brief, the notion of a Markov blanket allows one to define any system or structure in a way that distinguishes it from the environment or milieu in which it resides. The Markov blanket plays the role of a statistical boundary that allows one to talk about a system *per se* (Alcocer-Cuarón et al., 2014; Schrödinger, 1944). Such structures can be described at multiple scales, from macromolecules such as ribonucleic acids, through organelles to organs and organisms and even beyond. A Markov blanket is a set of states that separates the internal or intrinsic states of a structure from extrinsic or external states. Importantly, when interactions between states are spatially dependent, as is the case for states pertaining to the physical description of biological organisms, this separation can be spatial in nature. Consequently, in this setting, a Markov blanket describes a spatial boundary. Moreover, this boundary comprises sensory and active states, as is the case for biological systems, like membrane receptors and the cytoskeleton underneath them. In other words, a (biological) physical boundary is a Markov blanket (with sensory and active states), where dependencies between states are determined by location in space. And obvious example here would be the membranes that surround organelles and cells. The crucial aspect of a Markov blanket is that it provides a formal definition of what it means for the internal states of a structure to exist in a way that is conditionally independent of its external states. This definition precludes a (spatially dependent) direct coupling between internal and external states, such that they only influence each other vicariously through the Markov blanket (Friston 2013).

Markov blankets play a central role in several disciplines. For example, in Bayesian statistics and machine learning, they organise the architecture of message passing in neuronal networks and, indeed, the way we implement many statistical tests (Pellet and Elisseeff, 2008). In control theory, they underlie the circular interactions between system and environment (Baltieri and Buckley, 2018). In theoretical biology, they are the cornerstone of variational approaches to self-organisation under the free energy principle. These variational treatments have been applied at many levels; ranging from variational ethology and evolution (Ramstead, Badcock et al. 2017, Constant, Ramstead et al., 2018), through to self organisation, and adaptive behaviour in neuroscience (Friston 2010, Limanowski and Blankenburg 2013), down to morphogenesis and pattern formation at the cellular level (Kiebel and Friston 2011, Friston et al., 2015), and up to mental manipulation and imagination (Hohwy, 2016; Yufik and Friston, 2016). In this sense, the Markov blanket is a scale free concept that underwrites the dynamics of all self-organising systems, at some level.

Markov blankets are not necessarily spatially extensive membranes; they are just a set of states that separates internal and external states. For example, the brain's Markov blanket might include all its sensory receptors and neuromuscular junctions. At the cellular level, Markov blankets can be associated with membranes that surround cells and intracellular organelles, or to the surface of membrane-less organelles where a liquid-liquid phase separation takes place (Mitrea and Kriwacki, 2016). Notably, any spatially dependent interactions between system and environment can exist only in virtue of the permissive role of a statistical boundary; that is, a Markov blanket. However, this does not constrain such interactions within the physical boundary, such in the case of channels allowing ions to cross the cellular membranes or the production of heat by warm-blooded animals (Virgo, 2011; Virgo et al., 2011).

Previous work has already addressed the inextricable link between Markov blankets and living organisms. In particular, any biological self-organising system can be viewed as generating and maintaining Markov blankets at multiple scales (Friston, 2013). Consequently, morphogenesis at any particular level of description becomes the process of constructing a Markov blanket with a particular structure, as exemplified by the organisation of an ensemble of undifferentiated cells into a differentiated target morphology (Friston et al., 2015; Kuchling et al., 2019). In a companion paper, we have articulated the implications that the emergence of nested Markov blankets have for our understanding and interpretation of an organism's dynamics, with the important consideration that Markov blankets do not have to be co-extensive with the biophysical boundaries of an organism (Kirchhoff et al., 2018). These arguments are in turn tightly connected with considerations about a system's cognitive domain that exuberates spatial boundaries, analogously to the cognitive domain of a `glinder’ (a set of On states surrounded by Off states) in the Game of Life (Beer, 2014). In the present paper, we ask how an ensemble of constitutive parts, endowed with Markov blankets, could self-organise to create a Markov blanket at a higher scale; namely, a Markov blanket of Markov blankets. In particular, we focus on the minimal set of prior beliefs, a hierarchically organised system must express, and how these beliefs at different scales are linked.

Our basic conclusion is that a single principle is sufficient to explain the emergence of hierarchical structure; namely the variational free energy principle. This does not imply that hierarchical organisation is an emergent feature of any coupled random dynamical systems; rather, with the right sort of generative model, an ensemble of Markov blankets (e.g., cells) can self-assemble a Markov blanket around the ensemble (e.g., an organ). A generative model here refers to a probabilistic model of how external states influence the Markov blanket that is implicit in the dynamics of internal states. In the current setting, having the right sort of generative model can be regarded as having the right sort of prior (probabilistic) beliefs that are endowed by evolution. In what follows, we will use simulations to provide a numerical proof of principle that minimising variational free energy (under a suitable generative model) leads to hierarchical self-organisation. Throughout the paper ``free energy” will refer to variational free energy. While this is closely related to the thermodynamic concept of free energy (see Friston, 2019 for details), variational free energy is an informational quantity that provides an upper bound on surprise (a.k.a., surprisal or the negative log probability of sensory data).

This paper is organised as follows: first, we review the concept of a Markov blanket in biological systems and draw the link between statistical independence and physical boundaries. By doing so, we provide an intuition on the chief role that Markov blankets have in self-organisation within the free energy principle. We then consider the implications of the existence of a Markov blanket for the behaviour of random dynamical systems obeying the variational free energy minimisation principle. A technical treatment of Markov blankets in the emergence of physical structures and associated (quantum, stochastic, and classical) mechanics can be found in Friston, 2019). This section emphasises the autopoietic nature of systems that (Maturana, 1974), through the dynamics of their internal and active states, resist a natural tendency to disorder. In the final sections, we describe simulations of self-organisation at two levels: these furnish a proof of concept for self-organisation into Markov blankets and the hierarchical formation of blankets of blankets, respectively. We conclude with a discussion of how this treatment relates to other characterisations of biological self organisation.

## 2 Markov blankets and variational treatments of self organisation

Biological systems generally segregate themselves from their environment to form boundaries, which define the distinction between what is internal to the system and what is external (Alcocer-Cuarón et al., 2014; Kauffman, 1993; Kelso, 1995; Nicolis and Prigogine, 1977; Schrödinger, 1944). In this paper, these boundaries are formalised in terms of Markov blankets; namely, statistical boundaries that separate internal and external states (e.g., a cellular membrane separating intracellular and extracellular dynamics). In particular, spatial boundaries are an instantiation of the statistical independencies, when physical state interactions are spatially dependent, as is often the case for biological systems. This separation is a fundamental property of self-organising systems, because their very existence implies the presence of a boundary that distinguishes inside (i.e., self) from the outside (i.e., environment).

Living systems maintain the integrity of their boundaries (i.e. Markov blankets), in the face of an ever-changing environment. This means that life has evolved mechanisms for the generation, maintenance, and repair of Markov blankets. A system endowed with such mechanisms connotes an autopoietic organisation that autonomously assembles its own components; in particular its boundaries, (Maturana, 1974; Varela et al., 1974). This autonomy does not imply isolation from the environment, which – on a thermodynamic account – is needed to provide energy (Whitesides, 2002). Therefore, living organisms are operationally closed, while presenting as thermodynamically open. The interaction between system and environment is then mediated by the boundary. Notably, this coupling is non-trivial, in that the organism must actively realise an ‘informational control’ of the environment (i.e., possess a teleology), by filtering, canalising and categorising signals that carry information about their external causes (Auletta, 2010). This implies that the system does not merely respond to sensory states, but reacts to them to infer some (useful) information about the world. At the same time, the boundaries must contain machinery that allows the system to act on external states. In short, definitive borders are essential for living systems, as any dynamics that happens within and between systems can only take place in virtue of their existence (Friston, 2013).

Living organisms are complex systems, denoted by non-linear interactions between multiple hierarchically arranged and nested components (Hilgetag et al., 2000; Kauffman, 1995, 1993; Kirchhoff et al., 2018). As such, characterising how they self-organise requires not only an understanding of how single components couple to each other, but also how microscopic and macroscopic levels interact. This invokes the notion of top-down influences on the low level dynamics (Ellis et al., 2012) and vice versa.

Self-organisation has been addressed extensively in theoretical biology using tools from statistical thermodynamics and information theory to explain how biological systems resist a natural tendency to disorder. This holdout is an apparent violation of the second law of thermodynamics, or at least standard descriptions of it (Evans and Searles, 2002; Seifert, 2012). A more recent line of work within this framework (Friston 2013) sees living organisms as placing an upper (free energy) bound on their self-information (i.e., negative log likelihood of sensed states). This imperative is motivated by the fact that biological systems have to maintain sensory states within physiological bounds. This means the Shannon entropy (i.e., dispersion) of sensory states is necessarily bounded. Shannon entropy is the path or time average of self-information; also known as *surprisal* or *surprise*. In short, self-organisation can be regarded as synonymous with systems that place an upper bound on their self-information or surprise. In this variational formulation of self-organisation – that emphasises its inferential aspect – living organisms are understood as placing a (free energy) bound on surprise.

These arguments rest upon ergodicity assumptions (implicit in the fact that the sorts of systems we are interested in have characteristic measures that persist over time). Ergodicity implies that, over a sufficiently long period, the time spent in a particular location of state-space is equal to the probability that the system will be found at that location when sampled at random (Friston, 2013). If this probability measure is finite, it means that any system will revisit all its states (or their neighbourhoods) time and time again. It is this peculiar behaviour that underwrites self-organisation; namely, the existence of an attracting set of states that endow living systems with characteristic states that they visit time and time again.

The existence of an attracting set means that one can interpret the long-term average of surprise as the entropy of the systems sensory states. Crucially, because surprise is (negative) Bayesian model evidence, minimising free energy – defined as an upper bound on surprise – is the same as maximising a lower bound on the evidence for an implicit model of how sensory states are generated. In other words, the system can be regarded as a (generative) model of its environment (Conant and Ashby, 1970), and will look as if it is gathering evidence for its own existence. This has been called self-evidencing (Hohwy, 2016). It follows that – by minimising free energy – biological systems place an upper bound to the entropy of their sensations by inferring their causes; this is also known as *active inference* (Friston et al., 2010), and is closely related to formulations of the perception-action cycle in the life sciences, like embodied cognition (Clark, 2009), artificial intelligence (Ay et al., 2008), and cognitive neuroscience (Fuster, 2004). In short, *self*-organisation entails the bounding of *self*-information that can be cast as *self*-evidencing.

In what follows, we use mathematical and numerical analyses that build upon a free energy formulation of pattern formation (Friston et al., 2015). We start with subsystems whose dynamics possess a Markov blanket as an attracting set. We then integrate the system until it self-organises into a stable configuration. Subsequently, we extend the simulation to consider hierarchical systems; namely, configurations of configurations (i.e., blankets of blankets) that could, in principle, be extended indefinitely.^1^ These simulations were used to test the following hypothesis: if the maintenance of Markov blankets can be cast as self-evidencing, then self-organisation should be an emergent property (Kirchhoff et al., 2018) of subsystems that `believe’^2^ they participate in – or are enclosed by – a Markov blanket. Because Markov blankets are defined by conditional independencies, the requisite beliefs can be specified simply, in terms of communication or signalling between subsystems. In other words, it should be possible to reproduce hierarchical self-organisation by equipping subsystems with beliefs about how they influence – and are influenced by – other subsystems. The next section considers the formal basis of our simulations, based upon random dynamical systems and their probability density dynamics.

## 3 The Markov blanket partition

This section associates random dynamical systems with living organisms, where the states of a system stand for its internal states (e.g., intracellular states), its blanket states (e.g., receptors on a cell membrane and the actin filaments of the cytoskeleton) and external states (e.g., extracellular milieu). The systems under consideration are complex (i.e., non-linear and hierarchical) and organise independently of any applied or external gradient: we will see that such systems exhibit a process of pattern generation that lead to definitive boundaries (i.e. Markov blankets), defining internal states and their relationship with external states.

The notion of a Markov blanket was originally proposed in the context of Bayesian networks or graphs (Pearl, 1988), where it refers to the parents of the set of states (that influence it), its children (that are influenced by it), and the children's parents. The Markov blanket defines the conditional independencies between a set of states Λ (the system) and a second set of states Ψ (the environment). This concept can be translated into a biological setting: for example, the intracellular milieu of a cell represents the internal states and the plasmalemma corresponds to the Markov blanket, through which communication between intracellular and extracellular states is mediated (Auletta, 2013; Friston, 2013). Crucially, the Markov blanket can be decomposed into sensory *S* and active *A* states, which are and are not children of the external states, respectively. Thus, the existence of a Markov blanket *S* × *A* induces a partition of states in x∈X=Ψ×S×A×Λ; external states act on sensory states, which influence, but are not influenced by internal states. Internal states couple back through active states, which influence but are not influenced by external states (Table 1). This partition ensures a statistical separation between internal and external states in the sense they are independent, when conditioned on the Markov blanket.

**Table 1:** Definition of the tuple **Ω, Ψ, *S, A, M, p, q, P*** or self-evidencing.

*A sample space***Ω** from which random fluctuations ***ω*** ∈ **Ω** are drawn
*External states*$f_{\Psi }:\Psi \times A\times \Omega \overset{f_{\Psi }}{\to }\Psi \in R^{\Psi }$– states of the world (e.g. extracellular milieu) that depend on themselves and active states
*Sensory states*$f_{S}:\Psi \times A\times \Omega \overset{f_{S}}{\to }S\in R^{S}$– states of sensors (e.g. receptor activity) but depend upon external and active states
*Active states*$f_{A}:S\times M\times \Omega \overset{f_{A}}{\to }A\in R^{A}$ – states of action on the world (e.g. exocytosis of signalling molecules) that depend upon sensory and internal states
*Internal states*$f_{M}:S\times M\times \Omega \overset{f_{M}}{\to }M\in R^{M}$ – the internal states of a system (e.g. genetic transcription) that depend on themselves and sensory states
*Generative model****p***(***ψ, S, A, μ***|***m***) – a probability density function over external, sensory, active and internal states for a system denoted by ***m***
*Variational density****q***(***ψ***|***μ***) – a probability density function over external states parameterised by internal states

**Fig. 1:**
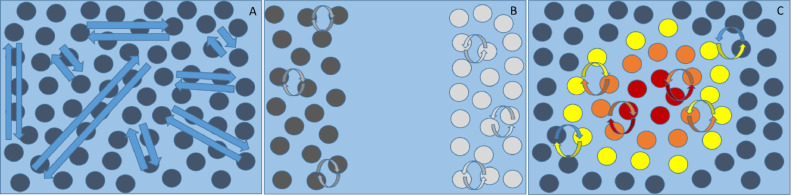
System comprising interacting states. In (A) spatially-independent coupling among states is mediated by long-range interactions. In the first (left) panel all states influence each other, and are therefore indistinguishable. In (B) only short-range interactions are allowed; thus coupling among states is spatially dependent. However, two sets of states exist only in virtue of their spatial separation: i.e., they are effectively independent. In (C), internal (red) and external (blue) states can be distinguished in virtue of the separation mediated by a third set; namely, the Markov blanket, composed of sensory (yellow) and active (orange) states. External states can influence internal states only by acting on sensory states. On the other hand, internal states couple back to external states through active states. Note that in this scenario, active states are shielded from external states by sensory states – and sensory states are shielded from internal states by active states. This is the simplest dependency structure leading to a Markov blanket. (For interpretation of the references to color in this figure legend, the reader is referred to the web version of this article.)

How this statistical concept can be translated into a biological setting, and why is its presence so important? We start by considering a system in which long-range (e.g., electromagnetic) interactions are possible, and states’ identity rests upon their coupling. Here, every state interacts with all others, irrespective of its spatial position; every state is therefore indistinguishable from the remainder, because the fully interconnected nature of the system precludes any statistical separation of one state from another (Fig. 1a). To engender statistical structure (i.e., an identity), coupling has to be limited via interactions restricted in space. One possible scenario displays two sets of states located far enough for them to be statistically independent: the state of one set does not influence the other and vice versa (Fig. 1b). However, in this case interactions between sets are also precluded. When considering biological systems and their environment, this scenario becomes unrealistic, given the definition of biological organisms as open systems (Whitesides, 2002). Therefore, two sets of states can be associated in a meaningful way to a biological organism's intrinsic (i.e. internal) and extrinsic (i.e. external) states only when a Markov blanket exists, which defines conditional or spatial independencies (i.e. identities) and interactions between the two states. Notably, it is the restriction in space of interactions that justifies the association between statistical and spatial independence, thus between Markov blankets and spatial boundaries. This is the minimal thus most general description of a biological self-organising organism possible (Fig. 1c). Notably, interactions occurs in virtue of the partition of the blanket states into sensory and active states, mediating the vicarious influence of external on internal states and the influence of internal on external states, respectively.

In reality, segregation emerges in the presence of coupling. In other words, a subsystem differentiates itself from the environment, but remains (statistically or energetically) coupled to it. This is possible when two sets of states are conditionally independent not just because of their spatial separation, but in virtue of a third set; namely, blanket states (Fig. 1c). These blanket states comprise sensory and active states, mediating the vicarious influence of external on internal states and the influence of internal on external states, respectively. This concludes our description of a minimal partition that enables a meaningful separation of internal and external states.

**Fig. 2:**
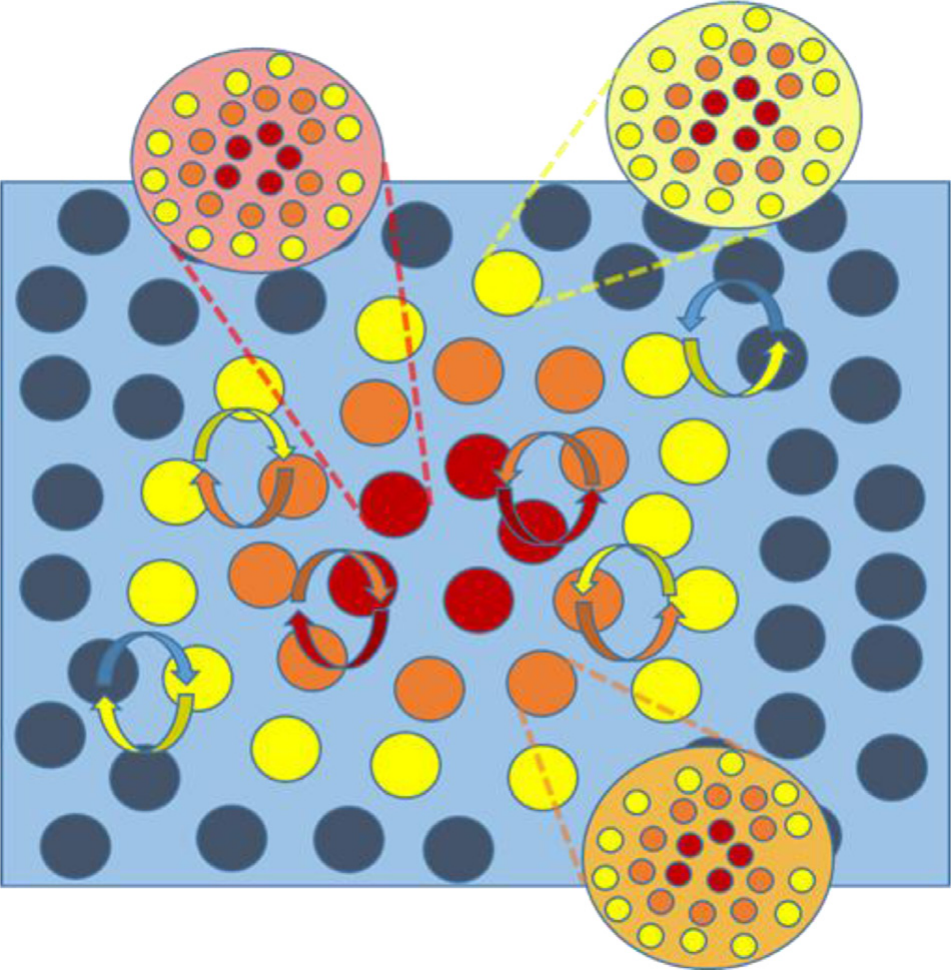
Markov blanket of Markov blankets. We now broaden the perspective, and consider each Markov blanket (and internal states) as a macroscopic state. Again, given short-range interactions, the only way for a system to exist at this macroscopic level is to be separated from its environment by a Markov blanket. The hierarchical nature of this system is induced by (macroscopic) Markov blankets of (microscopic) Markov blankets, each of them insulating its respective internal states. (For interpretation of the references to color in this figure legend, the reader is referred to the web version of this article.)

How can the concept of Markov blanket expand when considering the hierarchical structure of biological systems? Let us group internal and blanket states into a single (macroscopic vector) state. If this macroscopic state participates in some meaningful structure, a macroscopic Markov blanket has to emerge, whose sensory and active states – and the internal states insulated within – will each be composed of microscopic Markov blankets. Hence, the formation of Markov blankets at any level of hierarchical organisation is intimately linked to the maintenance of Markov blankets `all the way down’ (Fig. 2). On this view, self-organisation is a recursive process of boundary formation that spans all levels of hierarchical organisation. Later, we provide a proof of concept for this argument by simulating the hierarchical self-organisation of Markov blankets.

In summary, self-organisation has to feature the emergence of boundaries that define an internal state space, separating it from external states, while allowing for vicarious coupling. It follows that hierarchical self-organisation requires the emergence of Markov blankets of Markov blankets. In the next section, we turn to the nature of the dynamics that underwrite this emergence.

## 4 Dynamical systems, self-organisation and self-evidencing

We will be dealing with random dynamical systems expressed as Langevin equations of the following form:

(1) $$\begin{array}{c} \dot{x}=f\left(x\right)+\omega \\ f\left(x\right)=\left[\begin{array}{c} f_{\psi }\left(\psi ,s,a\right)\\ f_{s}\left(\psi ,s,a\right)\\ f_{a}\left(s,a,\mu \right)\\ f_{\mu }\left(s,a,\mu \right) \end{array}\right] \end{array}\}$$

This describes the dynamics of a system with a Markov blanket in terms of the flow *f*(*x*) of its states and random fluctuations *ω*. The flow of external *ψ* ∈ Ψ, sensory *s* ∈ ***S***, active *a* ∈ ***A*** and internal states *μ* ∈ ***M*** in the second equality, conforms to the dependencies implied by a Markov blanket (see Table 1). External states can only be influenced by internal states through their Markov blanket, and are therefore called hidden states, because they are hidden behind the Markov blanket. In the specific setting of biological systems, the partition in Eq. (1) relies on the spatial location of states, and identifies sensory and active states as components of spatial boundaries.

An alternative formulation of Eq. (1) is in terms of a Lagrangian, which allows us to describe the system's dynamics in terms of a gradient flow using the Helmholtz decomposition. This rests upon ergodic assumptions implied by the existence of an attracting set, called a pullback or random global attractor. Following Crauel et al. (1999), Crauel and Flandoli (1994) and Friston (2013), one can express the flow of states in terms of a divergence-free component and a curl-free descent on a Lagrangian *L*(*x*) that corresponds to the self-information or surprise associated with any state. This rests upon ergodic assumptions implying the existence of an attracting set (conditioned of the model) in the state-space, called a pullback or random attractor (Crauel et al., 1999; Crauel and Flandoli, 1994), and an associated probability density, the ergodic density.

(2) $$\begin{array}{c} f\left(x\right)=\left(Q-\Gamma \right)\nabla L\left(x\right)\\ L\left(x\right)=-\ln p\left(x|m\right) \end{array}\}$$

Here, diffusion tensor Γ is half the covariance of the random fluctuations, and *Q* is an antisymmetric matrix that satisfies $Q\left(x\right)=-Q{\left(x\right)}^{T}$. The equality p(x|m)=exp(−L(x)) is the solution of the Fokker-Planck equation describing the density dynamics (Friston and Ao, 2012; Frank, 2004), where *m* denotes a particular system or model (see Variational Free energy section). This ergodic or nonequilibrium steady-state density is the probability density at which its rate of change is zero. Eq. (2) means the states of a system *m* at nonequilibrium steady-state are performing a gradient ascent on the ergodic density. This is revealing, because it shows that the system's flow counters the dispersive effects of random fluctuations – by flowing towards the attracting states.

(3) f(x)=(Γ−Q)·∇lnp(x|m)

This gradient flow formulation also applies to the flow of internal and active states

(4) $$\begin{array}{c} f_{a}\left(s,a,\mu \right)=\left(\Gamma -Q\right)\cdot \nabla_{a}\ln p\left(s,a,\mu |m\right)\;\\ f_{\mu }\left(s,a,\mu \right)=\left(\Gamma -Q\right)\cdot \nabla_{\mu }\ln p\left(s,a,\mu |m\right) \end{array}\}$$

These equations are the homologues of (2) for the internal and active states, whose flow performs a gradient ascent on the ergodic density over the internal states and their Markov blanket (note that this density does not involve the external states, in virtue of the dependencies in Eq. (1)). In short, the internal and blanket states that constitute a subsystem are autopoietic, because their (nonequilibrium steady-state or ergodic) probability density is maintained by the flow of the subsystem's internal and active states. In the context of spatially dependent interactions, the flows partition expressed in Eq. (4), afforded by the Markov blanket formalism, relies on the spatial separation of states by a spatial boundary.

## 5 The variational free energy formulation

The flow of the states therefore describes a gradient ascent on the ergodic density. Analogously, in the setting of the stochastic thermodynamics, the system will minimise its thermodynamic free energy (Seifert, 2012). The link between thermodynamic and variational free energy rests upon associating the amplitude of random fluctuations on the motion of states with temperature – and equipping them with particular units through the use of Boltzmann's constant (Friston, 2019; Seifert, 2012; Sekimoto, 1998). This means that the changes in variational free energy inherent in belief updating can be linked directly to changes in thermodynamic free energy in a way that is consistent with the Jarzynski equality (Jarzynski, 1997) and Landauer's principle (Bennett, 2003; Landauer, 1961). Please see Friston (2019) for a fuller discussion and England (2013) and Parrondo et al. (2015) for a related perspective. Although the ergodic density exists, it is not evaluated explicitly by the system, because this would require access to external states that are hidden behind the Markov blanket. However, it is possible to use an alternative formulation that furnishes a description of the flow in terms of a gradient descent on a variational free energy associated with a generative model of the system in question (Friston et al., 2015):

(5) $$\begin{array}{c} \begin{array}{c} f_{\mu }\left(s,a,\mu \right)=\left(Q_{\mu }-\Gamma_{\mu }\right)\nabla_{\mu }F\\ f_{a}\left(s,a,\mu \right)=\left(Q_{a}-\Gamma_{a}\right)\nabla_{a}F \end{array}\;\\ F\left(s,a,\mu \right)=E_{q}L\left(x\right)-Hq\left(\psi \right)|\mu \end{array}\}$$

Here, the flow of internal and active states has been expressed as a gradient descent on variational free energy, which is a function of states that are available to the system. This follows because free energy depends on a variational density *q*(*ψ*|*μ*) over external states that is parameterised by internal states, and a generative model *p*(*ψ, s, a, μ*|*m*), which is the system itself, where *m* denotes the particular system (Friston et al., 2015).

Under this formulation of density dynamics, internal states will appear to *infer* external states: the third equality expresses free energy as the self-information (i.e., negative log evidence for the model) expected under the variational density minus the entropy of the variational density. This means that internal and active states maximise the joint probability density – expected under the variational density – over states conditioned on the system or model in question. Moreover, internal states will reduce free energy by parameterising a variational density over external states with maximum entropy; in accordance with Jaynes’ principle of maximum entropy (MacKay, 2003). Although not our focus here, when variational free energy is minimised, the variational density becomes the posterior density over hidden or external states, given blanket states. In this sense, the internal states encode posterior `beliefs’ about external states; despite never seeing them directly.

Crucially, the free energy formulation allows us to prescribe the ergodic density in terms of a generative model. In other words, we can write down a generative model and derive the dynamics according to Eq. (5) as a gradient descent on the free energy equivalent of surprise. In what follows, we will simulate self-organisation by specifying a model about the causes of sensory states – and by specifying the environmental dynamics generating those sensations. This means we need to write down the generative model *p*(*ψ, s, a, μ*|*m*) of the system in terms of the dynamics *f_ψ_*(*ψ, s, a*) and *f_s_*(*ψ, s, a*) of the environment and how sensory states are generated. Interestingly, the generative process and model do not have to be isomorphic: the generative model has only to approximate the generative process to minimise free energy (Baltieri and Buckley, 2018). The generative model is usually expressed in terms of random differential equations and nonlinear functions with a hierarchical form. In this paper, we will omit these dynamics for simplicity, and specify the relationship between external and sensory states through the following (static) nonlinear functions:

(6) $$\begin{array}{c} \begin{array}{c} s=g^{\left(1\right)}\left(\psi^{\left(1\right)}\right)+\omega^{\left(1\right)}\\ \psi^{\left(1\right)}=g^{\left(2\right)}\left(\psi^{\left(2\right)}\right)+\omega^{\left(2\right)} \end{array}\\ \vdots \end{array}\}$$

Under Gaussian assumptions about random fluctuations ω, Eq. (5) prescribes the likelihood and priors defining the generative model or Lagrangian:

(7) $$\begin{array}{c} p\left(\psi ,s|m\right)=p\left(s|\psi^{\left(1\right)}\right)p\left(\psi^{\left(1\right)}|\psi^{\left(2\right)}\right)\;\\ p\left(s|\psi^{\left(1\right)}\right)=N\left(g^{\left(1\right)}\left(\psi^{\left(1\right)}\right),\Pi^{\left(1\right)}\right)\\ p\left(\psi^{\left(1\right)}|\psi^{\left(2\right)}\right)=N\left(g^{\left(2\right)}\left(\psi^{\left(2\right)}\right),\Pi^{\left(2\right)}\right)\\ \vdots \end{array}\}$$

Here, Π^(*i*)^ corresponds to the precision or inverse variance of the random fluctuations. This allows us to completely specify the generative model in terms of beliefs about how sensations are generated and priors about hidden states. The key question we address in the next section is: what are the right priors that enable the emergence of Markov blankets at a higher macroscopic level – that would enable us to interpret the ensuing macroscopic dynamics in terms of the self-evidencing above.

In the simulations of subsequent sections, we integrate Eq. (5) using the Matlab routine **spm_ADEM.m** in the SPM open source academic software. This generalised Bayesian filtering scheme uses the Laplace assumption; i.e., the assumption that the variational density has a Gaussian form. The use of a Bayesian filtering scheme follows because the variational density *q*(*ψ*|*μ*) over external states approximates the posterior density *p*(*ψ*|*s, a, μ*): please see Friston (2014; 2010) for details. In summary, one can use standard Bayesian filtering to simulate self-organisation. This allows one to specify the form of the ergodic or nonequilibrium steady-state density in terms of the priors of a generative model. The question now is: what sort of priors leads to hierarchical self-organisation?

## 6 Self-organisation of an ensemble

In what follows, we present two sets of simulations. The first considers the self-organisation of an ensemble of synthetic cells, where each cell possesses its own Markov blanket. The second simulation considers ensembles of ensembles to illustrate hierarchical self-organisation; namely, the self-assembly of Markov blankets of Markov blankets. Crucially, these simulations use simple generative models, embodying the prior ‘belief’ that each member can play the role of an internal, active or sensory state within the ensemble. In other words, Markov blankets at one level of organisation possess prior beliefs there is a Markov blanket partition at the level above. However, each cell has no prior belief about its particular role in the higher level Markov blanket – or the form and composition of this blanket. These elementary priors are easy to specify because each role just depends upon the influences each member of the ensemble can or cannot exert on the others. This means, the only hidden state each member needs to infer is which role it plays at the higher level. We will see that this minimal set of prior beliefs (and subsequent self-evidencing) results in the formation of Markov blankets within the ensemble. The ensuing self-similar organisation can, in principle, be extended to any number of hierarchical levels. We illustrate this kind of hierarchical self organisation using 16 cells, each with their own Markov blanket, that organise into a cellular group or assembly, with its own Markov blanket. We then consider an ensemble of ensembles that organises itself into a little organ encompassed in another Markov blanket.

The first simulation illustrates the self-organisation of an ensemble. Each cell interacts with other cells; in a process that eventually leads to a stable configuration with a boundary separating internal cells from their external milieu. This simulation draws on previous work that interest morphogenesis (Friston et al., 2015). In this setting, self-organisation was simulated by minimising the variational free energy of each cell until they attained a prescribed morphology. This morphology was achieved through spatially dependent (e.g. chemical) signalling – so that every cell sensed every other cell in a way that was consistent with their generative models. The morphology was inscribed in beliefs common to all cells, about cell identity, sensation and secretion. Each cell was interpreted as a Markov blanket surrounding internal states: the action (active states) of a cell was the cause (i.e., external states) of the sensations (i.e., sensory states) of the remaining cells. At the beginning of pattern-formation, cells were undifferentiated, because they were uncertain about their identity in the target morphology. As self-organisation unfolded, each cell inferred a unique identity, location and what they should sense at that location. When every cell was in the right place, these inferences were fulfilled; thereby minimising the free energy (i.e., self information or surprise) of every cell.

In more detail, this inference – in analogy to intracellular cascade signalling and epigenetic mechanisms – was driven by the minimisation of free energy. By generating identity-dependent predictions (e.g. genetic and epigenetic expression) about sensations, every cell moved around and produced extracellular signals until its predictions were confirmed. Predictions about sensations caused by other cells (e.g. extracellular signalling) and its own action (e.g. secretion and position) were constrained by prior beliefs about the role of each cell in the target morphology. These prior beliefs were the same for every cell (c.f., pluripotential or stem cells). In other words, based on its identity, each cell had particular expectations about its sensory states. Because sensations were caused by other cells, surprise could only be minimised when every member of the ensemble had inferred a unique role within the ensemble. In short, priors established a point attractor for the ensemble dynamics, in terms of a free energy minimum, leading to differentiation and self organisation to a target morphology.

In the present work, we use the same strategy: we simulate self-organisation of an ensemble of cells, coupled through spatially decaying (e.g. chemical) signals. However, here, there is no target morphology – only the prior that every cell will play the role of an internal, sensory, or active cell, depending upon what it senses. In other words, the priors embody the conditional independencies implied by the existence of a Markov blanket; in the form of intracellular and extracellular signalling between three cell types. As the external states of each cell are the active states of other cells, the system organises in a pattern that enables each cell to predict signals from its companions as precisely as possible.

From the perspective of the ensemble there are no external states. This is an important point, as self-organisation is by definition autodidactic: it does not require coupling with an external environment. The ensuing process leads to a spatial pattern, wherein components of the system are organised in a predictable fashion with respect to each other. Such a pattern is inscribed in the (e.g., genetically encoded) prior expectations about the sorts of signalling a cell should expect to participate in. More precisely, priors are over parameters that specify the form of the generative model, which shapes the free energy landscape, thus defining the attracting states towards which the dynamics of the ensemble converge (Friston et al., 2015).

We now describe our simulation setup. The system comprised sixteen pluripotential cells, which can become one of three types of cells at the next hierarchical level; namely, internal, active and sensory cells. Each cell type secretes a unique extracellular signal and communicates according to the conditional independencies required by a Markov blanket (see Table 2). The external states of each cell comprised its location $\psi_{x}\in R^{2}$ and the chemical signals $\psi_{y}\in R^{3}$ released. This can be expressed as:

(8) $$\psi =\left[\begin{array}{c} \psi_{x}\\ \psi_{y} \end{array}\right]=\left[\begin{array}{c} a_{x}\\ a_{y} \end{array}\right]$$

**Table 2:** Prior beliefs characterising dependencies and independencies.

$p_{y}=\begin{array}{cc} \begin{array}{c} \begin{array}{ccc} \mu & a & s \end{array}\\ \left(\begin{array}{c} 1\\ 0\\ 0 \end{array}\;\begin{array}{c} \;0\\ \;1\\ \;0 \end{array}\;\begin{array}{c} \;0\\ \;0\\ \;1 \end{array}\right) \end{array} & \end{array}$	Prior probability matrix *p_y_* over sensed intracellular signals *S_y_*. Each cell secretes one of three types of signal.
$p_{\alpha }=\begin{array}{c} \begin{array}{c} \begin{array}{ccc} \mu & a & s \end{array}\\ \left(\begin{array}{c} 1\\ 1\\ 0 \end{array}\;\begin{array}{c} \;1\\ \;1\\ \;1 \end{array}\;\begin{array}{c} \;0\\ \;1\\ \;1 \end{array}\right) \end{array}\begin{array}{c} \begin{array}{c} \mu \\ a\\ s \end{array} \end{array} \end{array}$	Prior probability matrix *p_α_* over sensed extracellular signals *S_α_*. Sensory states (*s*) can interact with active states (*a*); active states can interact with internal (*μ*) and sensory states; sensory states can interact with active states; every cell exchanges of signals with cells of the same type.

Active states *a_x_* and *a_y_* (e.g., endoskeleton and secretory apparatus respectively) have an immediate effect on external states; hence the identity mapping. This simplifying assumption means we are ignoring time lags (and attenuation), and implies that there is no environment, other than elements of the ensemble contributing to the external states. Its sensory states are the sensed intracellular (produced by itself) and extracellular (produced by other cells) signals. The latter is a function of distance, assuming signal concentration decreases exponentially over space. This can be expressed as:

(9) $$s=\left[\begin{array}{c} s_{y}\\ s_{\alpha } \end{array}\right]=\left[\begin{array}{c} \psi_{y}\\ \alpha \left(\psi_{x},\psi_{y}\right) \end{array}\right]+\omega$$

Here, the sensory noise *ω* had a high precision (inverse variance) of exp(16). The sensed extracellular signals are returned by the function *α*(*ψ_x_, ψ_y_*), which models the spatial decay of signals, where the extracellular sensations of the *i*th cell are given by

(10) $$s_{i}=\alpha_{i}\left(\psi^{i},\psi^{j}\right)=\sum_{j}\text{exp}\left(-\left|\psi_{x}^{i}-\psi_{x}^{j}\right|\right)\cdot \psi_{y}^{j}$$

Here, *j* indexes all cells other than the *i*th cell. Each cell generates predictions based on the same generative model, which specifies the mapping from hidden states – namely, the *type* of the cell *ψ_i_* – to sensations. The type is then the only hidden state that the cells must infer. This inference is parameterised as an expected probability by their internal states *μ_i_*. Based on beliefs about its type, each cell then generates predictions about intracellular and extracellular sensations:

(11) $$\begin{array}{c} g\left(\mu_{i}\right)=\left[\begin{array}{c} p_{y}\\ p_{\alpha } \end{array}\right]\cdot \sigma \left(\mu_{i}\right)\\ \sigma \left(\mu_{i}\right)=\frac{\text{exp}\left(\mu_{i}\right)}{\sum_{i}\text{exp}\left(\mu_{i}\right)} \end{array}\}$$

Here, *p_α_* and *p_y_* are prior beliefs about secretion and sensation given the type of cell (see Table 2), generating sensory predictions, according to the generative model *g*(*μ_i_*) (see Eq. (6)), while *σ*(*μ_i_*) is a soft-max function that returns expectations about the cells type. The resulting dynamics of internal and active states of each cell can be expressed as follows:

(12) $$\begin{array}{c} f_{\mu }\left(\tilde{s},\tilde{a},\tilde{\mu }\right)=\left(Q_{\mu }-\Gamma_{\mu }\right)\nabla_{\mu }F=D\tilde{\mu }-\nabla_{\tilde{\mu }}\tilde{\varepsilon }\cdot \Pi^{\left(1\right)}\tilde{\varepsilon }-\Pi^{\left(2\right)}\tilde{\mu }\\ f_{a}\left(\tilde{s},\tilde{a},\tilde{\mu }\right)=\left(Q_{a}-\Gamma_{a}\right)\nabla_{\tilde{a}}F=-\nabla_{\tilde{a}}\tilde{s}\cdot \Pi^{\left(1\right)}\tilde{\varepsilon }\\ \Rightarrow \\ {\dot{a}}_{x}=-\nabla_{x}{\tilde{s}}_{\alpha }\cdot \Pi_{\alpha }^{\left(1\right)}{\tilde{\varepsilon }}_{\alpha }\\ {\dot{a}}_{y}=-\Pi_{y}^{\left(1\right)}{\tilde{\varepsilon }}_{y}\\ \varepsilon =\left[\begin{array}{c} \varepsilon_{y}\\ \varepsilon_{\alpha } \end{array}\right]=\left[\begin{array}{c} s_{y}-p_{y}\cdot \sigma \left(\mu \right)\\ s_{\alpha }-p_{\alpha }\cdot \sigma \left(\mu \right) \end{array}\right] \end{array}\}$$

Here ε=s−g(μ) is called a prediction error, and Π^(2)^ is the precision of a Gaussian prior over internal states that parameterise posterior beliefs about external states. The ~ notation denotes generalised coordinates of motion: see Friston, et al. (2010). The appearance of the precision-weighted prediction errors in this equation arises from the Laplace assumption alluded to earlier. Because variational free energy is expressed in terms of log probabilities, the Laplace assumption licenses a locally quadratic approximation to the variational free energy. As such, a second order Taylor series expansion around the posterior mode is sufficient to characterise this functional. Because the gradient of variational free energy evaluated at the posterior mode is zero (by definition), the linear term of this expansion vanishes. The result is that free energy may be expressed as a function that is quadratic in the distance between each variable and its posterior mode (i.e., quadratic in prediction errors). The gradient of the free energy is then simply expressed as a precision-weighted prediction error. Eq. (12) shows that internal and active states minimise (variational) free energy. Thus, internal and active states perform a descent to minimise prediction errors (Friston, 2010). Under these equations of motion, cells infer their identity based on sensations, while secreting according to their role as the ensemble evolves. At the same time, cells move to a position, where extracellular inputs can be best predicted.

Eq. (12) also evidences how surprise of observations given a particular model is reflected in the free energy. The term `surprise’ (a.k.a. surprisal) is used here in the information theoretic sense that quantifies how improbable a given event is (Tribus, 1961). Surprisal is a negative log probability (where the probability in question is the marginal likelihood of sensory states under the generative model). It is this quantity that is upper bounded by the free energy. Under the quadratic approximation to surprise (and variational free energy) employed in this paper, surprise scales with the squared difference between the expected and observed sensory state. This lets us associate surprise with a squared prediction error. As shown in Eq. (12), free energy is also a function of weighted squared prediction errors. As such, a surprising sensory state, when there is a mismatch between expected and observed data, leads to a large, precise, prediction error (*ε*) and an increase in free energy.

**Fig. 3:**
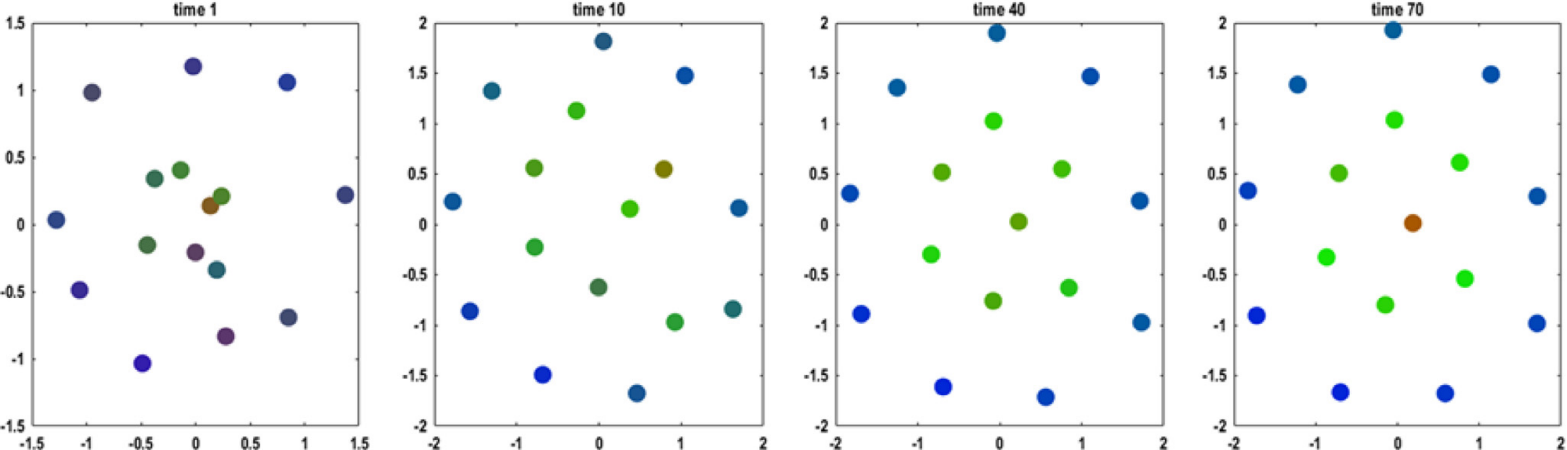
Self-organisation at the first level. This figure illustrates four snapshots at different times during the simulation of the (final stage of) self-organisation of an ensemble comprising sixteen ’cells’, whose internal and active equations of motion describe a gradient descent on prediction error, relative to sensory states expected by each member of the ensemble. Every member is endowed with the same prior (genetic) beliefs about what they should signal and sense, depending upon their type (which has to be inferred on the basis of what they sense). These priors ultimately prescribe a point attractor for the dynamics of the ensemble. Each cell can then infer (via intracellular dynamics) its type and behave (via extracellular signalling) accordingly, while moving (via chemotaxis) to a location that fulfils its predictions about its extracellular signals. The emergent morphology of the ensemble is a cell of cells, with an internal (red) cell in the centre, surrounded by a membrane of active (green) cells in the middle, and sensory (blue) cells on the periphery. This is the spatial pattern that best fulfils the prior beliefs of all the constituent cells. Note that the cells are initially pluripotent and only acquire (i.e., infer) their (colour-coded) role in the Markov blanket, in virtue of their position and signalling with other cells as they self-organise (see for an example the internal state at time 40 and 70). This means the number of sensory, active and internal cells is not encoded in each cell's prior; rather, it is an emergent property of self-organisation under the simple prior that each cell must be a particular type of cell. Furthermore, if a cell infers that it is a particular type, then it becomes that type – because its inference is mediated by intracellular signalling that classifies a cell as one type or another. (For interpretation of the references to color in this figure legend, the reader is referred to the web version of this article.)

The results of an exemplar simulation are shown in Fig. 3. Self-organisation leads the ensemble to assume a cell-like morphology, with internal cells in the middle, encircled by active cells, surrounded in turn by sensory cells. Because there are no prior beliefs either about the location or about the number of cells per type, these results constitute an emergent property, resulting from the spatial dependency of interactions among agents. In other words, the number of cells of each type is not pre-specified or part of the generative model – it is an emergent property. Furthermore, the arrangement of differentiated cells is not prescribed by each cell's prior beliefs – the arrangement is an emergent property that is consistent with intercellular signalling. This is interesting in the sense that the only arrangements that are consistent with a cell's beliefs about participating in a Markov blanket are exactly the arrangements that are consistent with a Markov blanket of cells: see also Cademartiri et al. (2012).

Although the results reported in Fig. 3 are sensitive to the priors that constitute each cell's generative model (please see discussion), they do not depend sensitively upon the initial states of each cell. In particular, random fluctuations in the initial positions and states of the cell do not affect the self organisation illustrated in Fig. 3. Exactly the same arrangement can be reproduced quantitatively with different initial randomisations. On the other hand, small changes to the priors, such as the spatial decay of extracellular signals – or the motility of cells – do affect the final configuration. For example, the number of internal cells can be greater than one and the final positions of the cells vary with different priors. Interested readers can repeat these simulations using different initial randomisations and prior settings, using open source code (please see Additional Material).

The simulation presented above illustrates the role of Markov blankets in a simple but plausible world where only local interactions are permitted, in which prior beliefs (e.g., a genetic code) have learned that, in order to exist, a living system has to self-generate boundaries that separate it from – and mediate the coupling with – its environment. As in real biological systems, the constituents of an ensemble interact with each other, leading to signal cascades. This signalling rests on inference (e.g., intracellular dynamics) about the role each cell should play, where action (e.g., chemotactic signalling) realises that role. Cells then differentiate, based upon their prior beliefs (e.g., genetic code). In essence, the ensemble reaches a steady state characterised by an internal milieu, which exists – in virtue of assembling its own Markov blanket – as integral part of the system. One could imagine that genes specify Markovian affordances to produce hierarchical structures; such as organs, tissues, organisms and so on. On this view, self-organisation is then a recursive process that engenders, at every level, the emergence of Markov blankets. We now pursue this using an extended simulation set up.

## 7 Self-organisation: ensemble of ensembles

The second simulation considers ensembles of organelles, each comprising an ensembles of 16 cells, to illustrate hierarchical self-organisation; namely, the self-assembly of Markov blankets of Markov blankets and the requisite coupling between levels. To investigate the autonomous organisation of (256) cells at two levels, every cell is equipped with the same (genetic beliefs or) priors about their local and global identity, that is, they share beliefs about possible roles at both the ensemble (local) and ensemble of ensemble (global) level. Practically, each cell now had two sets of hidden states – and prior beliefs – pertaining to their role at the local (i.e., microscopic) and global (i.e., macroscopic) level. Crucially, these priors are the same as used in the previous simulation; namely, they prescribe conditional independencies that are mandated by a Markov blanket at each level:

(13) $$\left[\begin{array}{c} p_{y}\\ p_{\alpha } \end{array}\right]=\left[\begin{array}{c} p_{y}^{l}\\ p_{\alpha }^{l} \end{array}\right]=\left[\begin{array}{c} p_{y}^{g}\\ p_{\alpha }^{g} \end{array}\right]$$

Here, the superscripts denote the local (ensemble) and global (ensemble of ensemble) level. The only additional piece of information required in this simulation is how the two levels couple to each other. For computational expediency, we model the microscopic dynamics (cells within an ensemble) of only one ensemble of sixteen cells, whereas for the remaining (fifteen) ensembles, we assume that the average behaviour conforms to the local dynamics of the simulated ensemble. This is a mean field approximation in the sense that we discount local fluctuations within each ensemble and assume only their average behaviour is ‘seen’ by any single ensemble. This allows us to simulate the coupling of sixteen cells of the fully simulated ensemble with other fifteen ensemble means (without simulating the other 15 ensembles explicitly). Notice that the allocation of cells to ensembles does not imply an allocation to a particular type of ensemble. The ensembles self-allocate as the Markov blanket emerges at the higher level. In summary, this simulation illustrates how sixteen cells self-organise in an ensemble that in turn self-organises with other fifteen identical ensembles, while describing the coupling between the local and global level.

In particular, for the fully simulated *k*th ensemble, the global to local extracellular coupling means that it only senses the average of all other global signals, while the local to global coupling means that the average over its *n* active states informs the dynamics of the remaining ensembles:

(14) $$\begin{array}{c} s_{\alpha ,\zeta }^{g}=s_{a,k}\\ s_{y,k}=a_{y,k}=\frac{1}{n}\sum_{\zeta }a_{y,\zeta }^{l} \end{array}\}$$

where ζ=1:n. The first and second equalities in (13) refer to the extracellular sensing of cells and the intracellular sensation of the ensemble, respectively. In terms of local to global coupling, as they are part of the same ensemble, these predictions will be congruent with each other and cells will therefore act in concert at the global level:

(15) $$g\left(\mu_{\zeta }^{g}\right)=\left[\begin{array}{c} p_{y}^{g}\\ p_{\alpha }^{g} \end{array}\right]\cdot \sigma \left(\mu_{\zeta }^{g}\right)$$

Here, $\mu_{\zeta }^{g}$ is the expectation about global coupling for each cell in the ensemble.

**Fig. 4:**
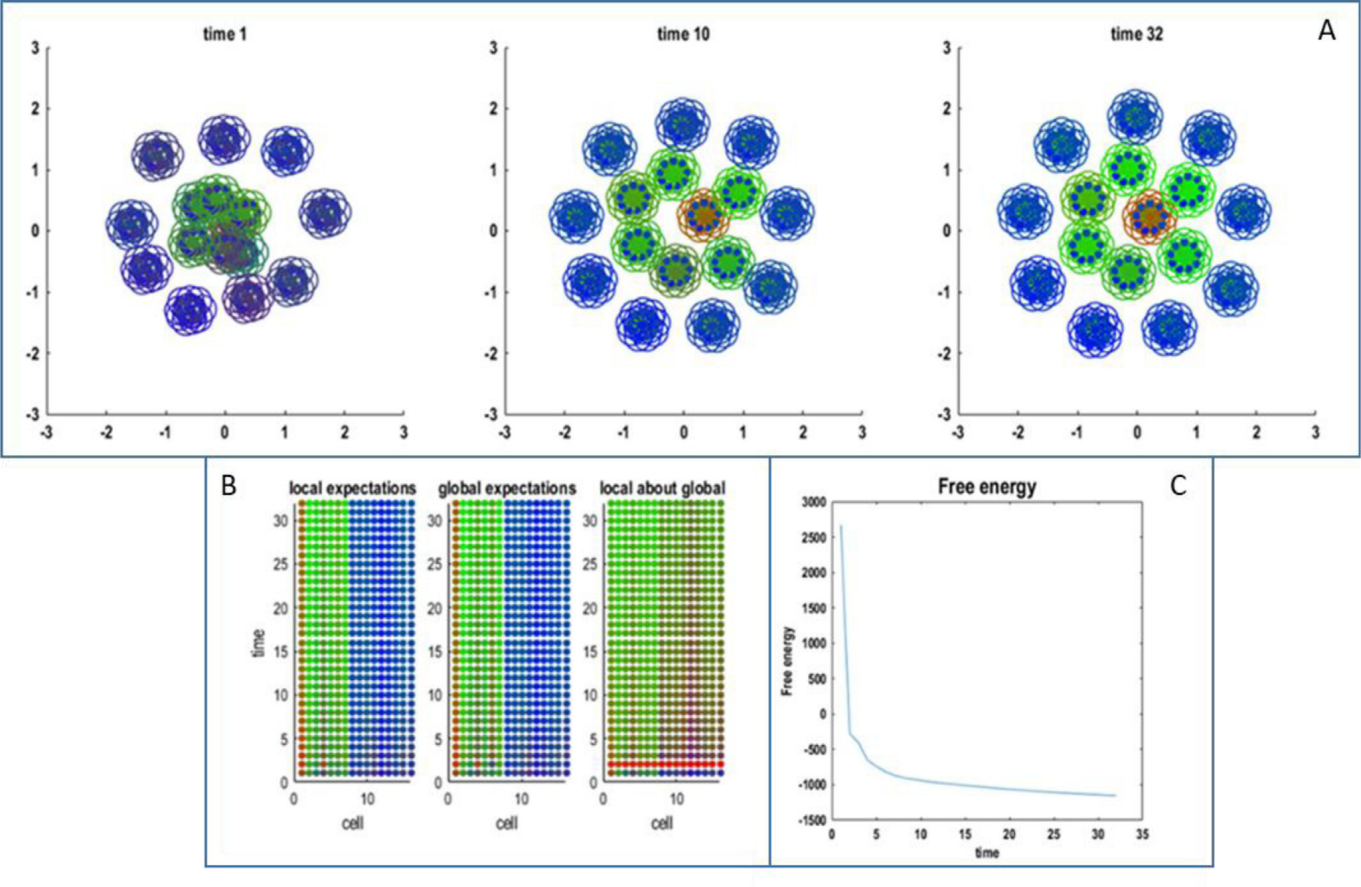
This figure shows the (final) results of self-organisation of an ensemble of cells, where each constituent of the ensemble is itself a local ensemble. In this example, 16 local ensembles, each composed of 16 cells, self-organise in a global ensemble. Panel A shows the evolution of the hierarchical system captured at three different moments. The colour of central circles reflects the inferred cells within each local ensembles; the peripheral circles indicate specialisation of local ensembles within the global ensemble. (colours: internal – red, active – green, sensory – blue). Note that there are no external states because the external states comprise the Markov blankets of other ensembles. The key thing to observe here is that (slow) self-organisation of local ensembles in a global Markov blanket, starting at time 1, relies on their very existence, that is, on (fast) self-organisation of cells composing each ensemble. At any temporal and spatial scale, the emergence of a Markov blanket reflects the particular independency structure, where internal cells do not influence sensory (i.e. surface) cells, in virtue of their separation by active cells. This separation induces conditional independence, because of the limited range of intracellular signals (that fall off with a Gaussian function of distance). Panel B shows the same results in an alternative format; namely, the evolution of expectations about type (i.e., differentiation) of cells within an exemplar local ensemble (left; local expectation), and of the local ensemble within the global ensemble (middle; global expectation). Notably, the identity of the local ensemble is the result (i.e. the average) of its constituent cell's beliefs about their role at the higher level. This means that a local ensemble organises in concert with the other in a global ensemble because its cellular components have communal beliefs about their role (as a local ensemble) in the global one. These cellular beliefs about global identity are represented in the left illustration of Pannel B. Here, the exemplar ensemble becomes an active state. This means that a local ensemble organises in concert with the others in a global ensemble because its cellular components have communal beliefs about their role (as a local ensemble) at the global level. These cellular ‘beliefs’ about global identity are represented in the left illustration of Panel B. Here, the exemplar ensemble becomes an active state. This means that all its constituents will come to infer that they participate as an active state at the global level (left; local about global). Note the differentiation on both a local and global level; while local expectations about the cells’ role at the global level converge to the same type. Panel C displays the decrease in free energy of the hierarchical system as self-organisation takes place. (For interpretation of the references to color in this figure legend, the reader is referred to the web version of this article.)

In summary, sixteen cells locally self-organise in an ensemble, guided by the (local) priors, while interacting with the remaining fifteen ensembles. Exemplar simulation results are shown in Fig. 4, which illustrates hierarchical self-organisation and pattern formation of Markov blankets within Markov blankets. The lower panels of Fig. 4 show the evolution of each cell's expectations (i.e., differentiation) at the local (left), and global (middle) level. The lower right panel shows the expectations of the cells of the sixth ensemble about their role at the global level. The sixth ensemble is an active ensemble at the global level (colour-coded by green), and this global identity appears to constrain the expectations of every cell at the local level.

A first interesting aspect of these simulation results is the rapidity with which the cells infer their type and implicitly differentiate into internal or blanket cells. This was a generic feature of the simulations, suggesting that the priors that lead to hierarchical self-organisation require a fairly rapid specialisation to enable self-assembly a global attractor. This reflects the circular causality or positive feedback loop between microscopic and macroscopic (Markov blanket) dynamics that underlies this kind of self-organisation. In other words, cells move and secrete chemicals accordingly to their inferred role in the Markov blanket partition; simultaneously, inference becomes more accurate as cells approach their final configuration. In the setting of hierarchical self-organisation, inference at the within ensemble (local) level is characteristically faster than at the between ensemble (global) level. This reflects a ubiquitous separation of temporal scales that characterises hierarchical self-organisation (Jung et al., 2015; Kiebel et al., 2008; Perdikis et al., 2011). A second noteworthy point is the spatial structure emerging in these exemplar simulations: when interactions are spatially dependent, active states – which can influence but are not influenced by external states – are enclosed by sensory states, in a similar way as the cytoskeleton resides within cellular boundaries, or muscles within the epithelium. Analogously, sensory states, that can influence but are not influenced by internal states, are segregated from the latter. Notice finally that as cells must possess a Markov blanket to distinguish each other at one level of description, at the level above there will be other ensembles (not shown) from which the simulated one will have to differentiate itself – by means of a Markov blanket.

In the example shown in Fig. 4, local expectations (lower left panel) converge quickly, with a discernible differentiation after the first time step. Conversely, local expectations about the global type take much longer to converge, with the degree of uncertainty in some cells that only resolves at the final time step (see cell 12 in the lower right panel). This example may reflect the separation of timescales formalised in synergetics, and in particular by the slaving principle, which deals with self-organisation and pattern formation in open systems far from thermodynamic equilibrium (Carr, 1981; Ginzburg, 1955; Haken, 1978; Tschacher and Haken, 2007). In this setting, slow macroscopic patterns of activity are said to enslave fast microscopic patterns, while the macroscopic patterns (known as order parameters) are constituted by the microscopic patterns; hence circular causality.

## 8 Discussion

In this paper, we have presented a variational treatment of hierarchical self-organisation. Given local interactions, carefully crafted prior (genetic) beliefs about conditional dependencies and independencies endow a system with a point attractor comprising internal states and their Markov blanket. Moreover, applying the same priors at any hierarchical level leads to the emergence of Markov blankets within superordinate Markov blankets. A key feature of the simulations – used in this paper – is the absence of any explicit target morphology within the prior (e.g., genetic) beliefs of the system's denizens. This is an emergent property, which appears to have a top-down effect on the blankets below. The subsequent emergence of a cell-like structure is interesting because it speaks to characteristic spatial boundaries found in most biological systems; namely, cellular membranes. The isomorphism between a statistical and spatial boundary rests on spatially dependent interactions among internal and external states. In other words, the states of a system can include its generalised motion in physical space, such that the blanket states acquire the attribute of a spatial location (and velocity). In turn, this means that spatial boundaries can be identified with statistical boundaries, under conditional dependencies of the flow internal and active states on external states. As noted above, these normally involve short range statistical couplings or, in physical terms, forces or chemical gradients (Ao, 2009; Seifert, 2012). Crucially, this sort of hierarchical self-organisation is a recursive process that can repeat itself at higher levels of description. Interestingly, this perspective enables to identify ensembles that are or are not self-organising: in this optic, the distinction between a culture of cells and a multicellular organism resides in the emergence of a Markov blanket at the ensemble level. Another consequence of this recursive aspect is the absence of a privileged point of view, when describing hierarchical self-organisation: the dynamics at every level play the role of macroscopic states at the level below, and the role of microscopic states at the level above. An apparent example is morphogenesis, during which chemotaxis and development of a system's components occurs within a global configuration established by morphogen gradients controlling gene expression (Balaskas et al., 2012; Chang et al., 2002). Notably, in our simulations, morphogen gradients could be interpreted as the expression of beliefs about the possible role at the ensemble level that each cell or element at the level below must have. Actuation of these beliefs (morphogenesis) then occurs through an inference process (differentiation), and realisation of the corresponding positional predictions (chemotaxis); for an example that uses exactly the notion of a Markov blanket and chemotactic signalling, please see (Kuchling et al., 2019). At a subcellular scale, the same logic applies, so that self-organisation of microscopic organelles like the cellular membrane and vesicular structures is constrained by beliefs about their role at the cellular level. On the same note, the dialectic between hierarchical layers formalised above accounts for the dynamics of multi-agent, complex systems, ranging from cultural ensembles (Ramstead et al., 2018) to complex urban environments (Hadfi and Ito, 2016).

Here we associate biochemical structures, gradient flows and kinetics with Bayesian priors and belief propagation or updating – as opposed to propositional or representational beliefs. The only thing that licenses the use of the word ‘belief’ is that the macromolecular and cellular kinetics at hand were cast as a gradient flow on variational free energy. This means that one can interpret the resulting dynamics in terms of Bayesian belief updating (Winn and Bishop, 2005). However, this is an `as if’ interpretation; in the same sense that the folding of a macromolecule to minimise its thermodynamic free energy in computational chemistry – e.g., Lammert et al. (2012) – looks `as if’ it is trying to minimise (thermodynamic) free energy.

The advantage of being able to formulate this kind of self-organisation in terms of gradient flows on variational (as opposed to thermodynamic) free energy is that variational free energy is a functional of a generative model. This means that one can prescribe the desired endpoint of self-organisation in terms of priors (Friston et al., 2014). One might ask; where do priors come from? Here, we assume that they are a product of natural selection (i.e., Bayesian model selection) and are therefore entailed in genetics and epigenetics (Campbell, 2016; Frank, 2012; Ramstead et al., 2018). We do not try to provide a detailed account of the ensuing gradient flows in terms of molecular biology; e.g., Tabata and Takei (2004). However, one can imagine hypotheses based on variational free energy gradient flows can be tied to intra-and inter-cellular signalling; e.g., Cervera et al. (2019) and Friston et al. (2015) and Kuchling et al. (2019). Interestingly, exactly the same challenge arises in the neurosciences, where the equivalent gradient flows become neuronal dynamics – and the accompanying challenges become understanding neurophysiology and neuronal microcircuitry in terms of variational message passing; e.g., Friston et al. (2017).

It is interesting to ask how the priors that underwrite this kind of self-organisation are updated in terms of cell biology. The usual response to this is to consider hierarchal processes of free energy minimisation in terms of Bayesian model selection (Allen, 2018; Campbell, 2016; Frank, 2004; Ramstead et al., 2018). This leads naturally to a link between natural selection and Bayesian model selection based upon the evidence bounds afforded by variational free energy. On this reading, natural selection becomes a form of structure learning (a.k.a. Bayesian model selection) based upon the model evidence associated with a particular phenotype. In short, the (marginal) likelihood of a particular phenotypic structure – in an evolutionary setting – is optimised in terms of its prevalence in a population. Because this structure is a model of the external milieu, it entails particular priors. If these priors are fit for purpose in terms of minimising variational free energy then they will be selected. For example, under some mild assumptions, the replicator equation can be cast as a Bayesian filter; exactly along the lines of the above argument: see Frank (2012), Geisler and Diehl (2003) and Ramírez and Marshall (2017) for further discussion.

In our simulations, and more generally, we have made some mild assumptions about the external or environmental states that contextualise self-organisation at the highest scale considered. This speaks to an important conceptual point; namely, that a partitioning of systemic states into Markov blankets at any scale is always contextualised by the scale above. In other words, there must be a permissive context in which self-organisation unfolds at, generally, faster timescales in the level below (Schwabl, 2002; Jeffery et al., 2019). Strictly speaking, this implies an infinite regress; in the sense that we can only talk about Markov blankets at one scale of self-organisation by assuming some attracting set at a higher scale. Indeed, this recursion can be formalised in terms of the renormalisation group that emerges from grouping and course graining (i.e., reduction) operators on the Markov blankets (Friston, 2019). The renormalisation group formulation implies that the time constants of self-organisation at larger scales necessarily increases, when moving from one scale to the next.

Practically, this means that one can assume that the external states that encompass the formation of Markov blankets at the highest scale under consideration are changing slowly in relation to lower scales. The picture that emerges here is that the same basic (Bayesian or variational) mechanics emerge in a scale-free fashion at different levels; from the quantum through to the level of molecular biology; from the scale of cells through to organs, from phenotypes through to species; all the way up to a cosmological scale. Although this might sound fanciful, this perspective has some currency in relation to the differences between quantum, statistical and classical mechanics. These differences rests largely upon the suppression of random fluctuations as one progresses from the small to the large. We have chosen to illustrate coupling between just two levels; namely, the mesoscopic level of cells and cell assembly in biology.

One might ask why we have focused on the free energy principle, as opposed to other formal descriptions of self-organisation (Haken, 1978; Kauffman, 1993; Kelso, 1995; Nicolis and Prigogine, 1977); for example, phase transitions in spin models (Vatansever and Fytas, 2018), attractor landscapes in random Boolean networks (Gershenson, 2012) or Turing style pattern formation via reaction diffusion systems (Halatek et al., 2018). Our motivation for casting self-organisation as a variational principle was threefold: first, the free energy principle provides an integrative formalism that should apply to all the above. In other words, it regards pattern formation and self organisation – as manifest in reaction diffusion systems and other nonequilibrium steady-state dynamics – as realisations of the same principle. This is self-organisation to a random attractor (Crauel and Flandoli, 1994; Friston and Ao, 2012). When this attracting set possesses a Markov blanket the free energy principle must apply (Friston 2013). This means that one can interpret any form of self-organisation – to an attracting set – in terms of a gradient flow on variational free energy and, implicitly, self-evidencing (Hohwy 2016, Ramstead, Badcock et al. 2017). This is important because most existing approaches to the dynamics of self-organisation try to reverse engineer an energy functional (or Lyapunov function), given some dynamics or equations of motion. The free energy principle allows one to invert the problem and write down the dynamics as a gradient flow on a free energy functional that is specified in terms of a generative model (Friston et al., 2014). Crucially, the prior beliefs of this generative model determine the attracting set. By explicitly writing down a Markov blanket in these priors, we obtain a system whose organisation is the most general and essential possible, implicitly in any self-organising system behaving accordingly to the free energy principle. Finally, using the notion of random dynamical systems (Arnold and Crauel, 1991; Crauel and Flandoli, 1994), the free energy principle allows one to articulate questions about (and simulate) self-organisation at multiple scales. Here, we have focused on the link between just two scales; however, by induction, the conclusions from this paper could be generalised to multiple levels – at least in principle. This hierarchical aspect would be challenging to simulate using conventional approaches to pattern formation.

As noted in the introduction, our aim was to provide a numerical analysis of the minimal conditions under which hierarchical self-organisation emerges – and show that the minimisation of variational free energy provides a sufficient account, *under the right sort of generative model*. Crucially, this does not mean that any free energy minimising ensemble will show this kind of hierarchical self organisation. Our agenda was not to suggest all systems self-organise hierarchically; rather, we wanted to explain the existence of the hierarchical structures seen in biology, in terms of variational principles.

Practically, the behaviour illustrated in the above simulations depends sensitively on priors in the generative model and initial conditions. In more details, setting prior beliefs that specify an attractor in state space (endowed with a Markov blanket) is not trivial, and growth in the size of the system further complicates the task. This is reminiscent of the emergence – at later evolutionary stages – of bigger or more complex organisms. Furthermore, these simulations show the final stage of self-organisation, where the system finds itself in the vicinity of the attractor; initialising the system too far from this attracting point (e.g., by adding extra perturbation to location) can prevent the system from elaborating a bounded structure (i.e. existing). This, in turn, speaks to the difference between these (minimal and general) simulations and the complexity of (specific) biological systems, endowed with a plethora of control and feedback mechanisms, which underwrite robustness to perturbations. This sensitivity to prior parameters and initial states leads to some interesting questions. For example, questions about the rate at which the structures stabilises – and how this depends upon the parameters (c.f., rate spatial decay constants) that constitute each cell's priors. How do the initial values (e.g., position) affect self-organisation? These questions raise an interesting issue: is there anything special about a hierarchical structure that would explain its prevalence in biotic systems. One speculation here might be that a hierarchical (self-similar) architecture of Markov blankets might be a free energy minimising solution on a longer time scale, such as evolution. This should be possible to address via simulated (pharmacological) lesion experiments that block the formation of higher-order Markov blankets. One can then measure the free energy with and without hierarchical self-organisation and consider the implications for natural selection. In variational formulations, natural selection is treated as a form of Bayesian model selection, based upon model evidence or variational free energy (Campbell, 2016; Frank, 2012). We hope to pursue this in subsequent work.

## 9 Conclusion

This work suggests that Markov blankets are a fundamental characteristic of biological systems. Their presence is necessary for life – as they underwrite an existential separation of the system from its environment, while preserving its interactions. The hierarchical organisation of complex systems – like living organisms – implies that the self-similar organisation of Markov blankets may be evident at any level of biological structure. From the point of view of dynamical systems, Markov blankets are attractors, attracting fast microscopic dynamics, while underwriting the emergence of macroscopic (order) parameters. This circular causality nicely captures the self-organisation of biological systems, which evolve autonomously with a morphology (Markov blanket) that is necessarily predisposed to a selective coupling with external states. The natural place – where these attractors might be specified – is the genetic code. Clearly, this is rather speculative; however, it is possible that the astonishing diversity of flora and fauna we witness might reflect the fact that, in a world where signals are spatially dependent, Markov blankets are synonymous with existence.

## Additional information

**Simulations:** The simulations reported in this paper can be reproduced using the open access academic software SPM (http://www.fil.ion.ucl.ac.uk/spm/software/). The key routines are **DEM_cells.m** and **DEM_cells_cells.m** that illustrate self-organisation of a single ensemble and ensemble of ensembles respectively.

**DEM_cells.m:** This demo illustrates self-organisation in an ensemble of (sixteen) cells using the same principles described in **DEM_morphogenesis**, but using a simpler generative model. Overall, the dynamics of these simulations show how one can prescribe a point attractor for each constituent of an ensemble that endows the ensemble with a point attractor to which it converges. In this example, we consider the special case where the point attractor is itself a Markov blanket. In other words, cells come to acquire dependencies, in terms of intracellular signalling, that conform to a simple Markov blanket with intrinsic or internal cells, surrounded by active cells that are, in turn, surrounded by sensory cells. This organisation rests upon intracellular signals and active inference using generalised (second-order) variational filtering. In brief, the hidden causes driving action (migration and signalling) are expectations about cell type. These expectations are optimised using sensory signals; namely, the signals generated by other cells. By equipping each cell with prior beliefs about what it would sense if it was a particular cell type (i.e., internal, active or sensory), they act (i.e., move and signal) to behave and infer their role in an ensemble of cells that itself has a Markov blanket. In a **DEM_cells_cells.m**, we use this first-order scheme to simulate the hierarchical emergence of Markov blankets; i.e., ensembles of cells that can be one of three types at the local level; independently of their time at the global level.

**DEM_cells_cells.m:** This demo is a hierarchical extension of **DEM_cells.m**, where we have 16 ensembles comprising 16 cells. Each cell has a generative model (i.e., prior beliefs) about its possible local and global cell types (i.e., internal, active or sensory). Given posterior beliefs about what sort of self it is at the local and global level, it can then predict the local and global intracellular signals it would expect to receive. The ensemble of ensembles then converges to a point attractor; where the ensemble has a Markov blanket and each element of the ensemble comprises a cell – that is itself a Markov blanket. The focus of this simulation is how the local level couples to the global level and vice versa. For simplicity (and computational expediency) we only model one ensemble at the local level and assume that the remaining ensembles conform to the same (local) dynamics. This is effectively a mean field approximation, where expectations of a cell in the first ensemble about its global type are coupled to the corresponding expectations and the ensemble level, and vice versa. The results of this simulation are provided in the form of a movie and graphs.

## Author contribution

Ensor Rafael Palacios and Karl Friston conceived the ideas, wrote the manuscript and performed the simulations. Adeel Razi conceived the ideas, reviewed the manuscript text and contributed to the simulations. Thomas Parr and Michael Kirchhoff reviewed the manuscript.

## CRediT authorship contribution statement

**Ensor Rafael Palacios:** Conceptualization, Methodology, Writing - original draft, Visualization. **Adeel Razi:** Conceptualization, Writing - original draft. **Thomas Parr:** Writing - original draft. **Michael Kirchhoff:** Writing - original draft. **Karl Friston:** Conceptualization, Methodology, Writing - original draft, Visualization.

## Declaration of Competing Interest

No competing financial interests to report.

## Appendix Supplementary materials

Image, application 1

## Footnotes

1 We thoroughly explore the implications of hierarchical organisation of Markov blankets for description of living organisms in the more philosophically motivated paper by Kirchhoff et al. (2018). On the other hand, from an evolutionary perspective, these arguments encourage new questions and interpretations, like the identity of the very first prior or major evolutionary transitions of priors in unicellular and multicellular organisms (see discussion).

2 This term is used as a synonym of prior expectations in the context of Bayesian inference.
